# Clearance of senescent hepatocytes in a neoplastic-prone microenvironment delays the emergence of hepatocellular carcinoma

**DOI:** 10.18632/aging.100631

**Published:** 2014-01-23

**Authors:** Fabio Marongiu, Maria Paola Serra, Marcella Sini, Fabrizio Angius, Ezio Laconi

**Affiliations:** Department of Biomedical Sciences, Unit of Experimental Medicine, University of Cagliari, 09124 Cagliari, Italy

**Keywords:** cell transplantation, tumor microenvironment, cell senescence, cell competition, liver repopulation, liver carcinogenesis

## Abstract

Increasing evidence indicates that carcinogenesis is dependent on the tissue context in which it occurs, implying that the latter can be a target for preventive or therapeutic strategies. We tested the possibility that re-normalizing a senescent, neoplastic-prone tissue microenvironment would exert a modulatory effect on the emergence of neoplastic disease. Rats were exposed to a protocol for the induction of hepatocellular carcinoma (HCC). Using an orthotopic and syngeneic system for cell transplantation, one group of animal was then delivered 8 million normal hepatocytes, via the portal circulation. Hepatocytes transplantation resulted in a prominent decrease in the incidence of both pre-neoplastic and neoplastic lesions. At the end of 1 year 50% of control animals presented with HCC, while no HCC were observed in the transplanted group. Extensive hepatocyte senescence was induced by the carcinogenic protocol in the host liver; however, senescent cells were largely cleared following infusion of normal hepatocytes. Furthermore, levels of Il-6 increased in rats exposed to the carcinogenic protocol, while they returned to near control values in the group receiving hepatocyte transplantation. These results support the concept that strategies aimed at normalizing a neoplastic-prone tissue landscape can modulate progression of neoplastic disease.

## INTRODUCTION

Population The role of the microenvironment in the pathogenesis of neoplastic disease is increasingly being appreciated. Starting from the report of Mintz and Illmensee [1], describing the generation of normal genetically mosaic mice from malignant teratocarcinoma cells, several studies have demonstrated that the phenotype of pre-neoplastic and neoplastic cell populations can be profoundly modulated by external cues emanating from the surrounding microenvironment [2-5]. Furthermore, it has been documented that specific gene-expression profiles in non-cancerous tissue are able to predict recurrence and survival in patients with hepatocellular carcinoma (HCC), again pointing to the critical role of the surrounding microenvironment in the natural history of neoplastic disease [6-7]. Along this line, studies from our laboratory have indicated that a growth-constrained/senescent tissue environment is able to generate a powerful driving force for the selective expansion of pre-neoplastic hepatocytes in the liver, leading to their progression to HCC [8]. Exposure to retrorsine (RS), a naturally-occurring pyrrolizidine alkaloid, impairs liver regeneration and induces extensive hepatocyte senescence in rat liver [9-10]. When pre-neoplastic cells isolated from hepatic nodules were transplanted in RS-treated livers, they grew rapidly and evolved into HCC; however, the same cell preparation was unable to expand and progress following injection into untreated, syngeneic normal hosts [8].

These observations provide a rationale for the hypothesis that targeting a neoplastic-prone tissue landscape may represent a valuable approach to modulate the evolution of carcinogenic process [11-13]. Recently, we have obtained evidence to indicate that orthotopic transplantation of normal hepatocytes in animals previously exposed to a carcinogenic regimen exerts a delaying effect on the growth of early preneoplastic lesions [14]. In the present studies, we have extended this observation and explored the possible biological and molecular mechanisms underlying this phenomenon. Neoplastic process was induced in rat liver through sequential exposure to diethylnitrosamine (DENA) and RS. Normal hepatocytes transplanted following the carcinogenic protocol were able to reduce the incidence of preneoplastic and neoplastic lesions at the end of 1 year. This was associated with clearance of RS-induced senescent hepatocytes by transplanted normal cells.

## RESULTS

### The induction of hepatocellular carcinoma following exposure to DENA+RS

**Figure 1:**
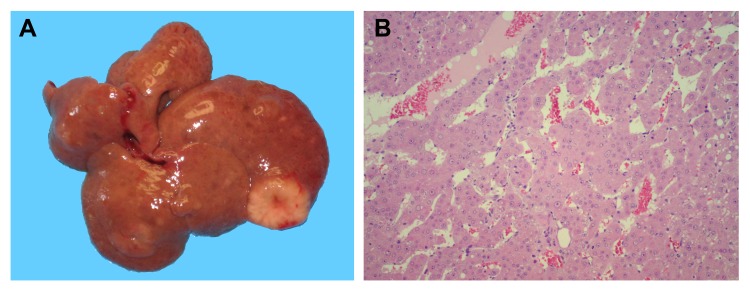
The development of HCC in rats exposed to DENA+RS and killed after one year. Panel **A**: macroscopic appearance, with withish-grey lesions displaying prominent vasculature; panel **B**: trabecular HCC with discrete cellular pleomorphism (100x).

As already mentioned, naturally occurring pyrrolizidine alkaloids, including RS, are known for their ability to promote the growth of early hepatic nodules in initiated rat liver [15]. However, no studies have been reported to date on the long term effects of these agents in animals previously given a carcinogen. In the present experiments, rats were administered DENA and RS (two single injections, 10 days apart), and they were killed 1 year later. As predicted, multiple pre-neoplastic and neoplastic hepatocellular lesions, ranging in size from a few mm to 2.5 cm in diameter, were observed in all animals exposed to this protocol (figure 1, panel A). Furthermore, histological analysis confirmed the pre-sence of large, advanced hepatocyte nodules in all liver samples in this group, while trabecular HCC was diagnosed in 4 out of 8 rats (figure 1, panel B).

### Normal hepatocyte transplantation delays the emergence of HCC induced by DENA+RS

**Figure 2:**
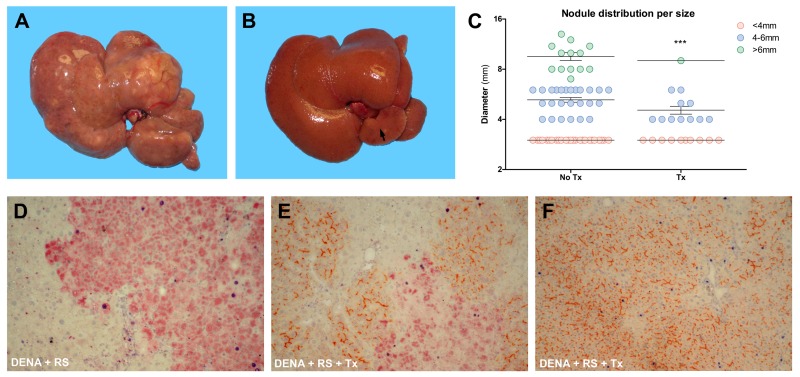
Analysis of liver lesions Macroscopic appearance of livers from animals exposed to either DENA+RS (panel **A**) or DENA+RS followed by hepatocyte transplantation (panel **B**); both animals were killed 1 year post-treatment. Note the presence of large lesions in panel **A**, while the liver in panel **B** appears normal and shows only one tiny nodule in the caudate lobe. Panel C shows the size distribution of hepatic lesions in both experimental groups; note that the largest lesion found in one animal in DENA+RS-treated group is not included in this plot. ***Significantly different from non-transplanted animals: nodules <4mm, P<0.005; nodules 4-6mm, P<0.001; nodules >6mm, P<0.005. Panels **D-F**: immunohistochemical analysis of liver sections from animals exposed to either DENA+RS (panel **D**) or DENA+RS followed by hepatocyte transplantation (panels **E** and **F**); sections were stained for glutathione-S-transferase 7-7 (GST 7-7, a marker of preneoplastic nodules), BrdU and DPP-IV (orange-rust). Note the presence of BrdU-labelled hepatocytes (dark blue) in GST 7-7-positive lesions (red color, panels **D** and **E**) and in areas of repopulated liver (orange-rust, panel **E** and **F**).

Based on the above findings, we next considered the effect of normal hepatocyte transplantation on the incidence of hepatic nodules and HCC following exposure to DENA+RS. Results are reported figure 2 and table 1. Major differences were already evident upon macroscopic examination. The liver of DENA+RS-treated animals displayed slightly increased stiffness compared to normal, with irregular margins and finely granular surface; however, these changes were largely reversed in rats receiving the infusion of normal cells (figure 2, panels A and B). Most notably, the presence of large nodular lesions and overall tumour burden in the liver were greatly reduced in the latter group (figure 2, panel C); only 2 out of 8 rats in this group had nodules >5mm in diameter; strikingly, in 2 animals no macroscopic lesions were observed. Histological analysis on H&E stained liver samples confirmed and extended these results: overt HCC was found in 4 out of 8 animals given DENA+RS, as mentioned in the preceding paragraphs; however, no HCCs were present in the group receiving normal hepatocyte transplantation following the carcinogenic protocol (table 1). Proliferating hepatocytes were readily observed in hepatic nodules and HCC in animals exposed to DENA+RS, as expected (figure 2, panel D); however, they were fewer in GST 7-7-positive lesions from the group receiving hepatocyte transplantation (figure 2, panel E); in the latter group, areas of repopulated liver displayed scattered BrdU-positive hepatocytes (figure 2, panel F).

**Table 1:** Incidence of nodules and HCC in the two experimental groups

	Number of animals with:		
	Preneoplastic nodules		HCC
	*≤5mm*	*>5mm*	
DENA + RS	8/8	7/8	4/8
DENA + RS + Tx	6/8	2/8	0/8
Relative Risk	0.7500	0.2857	0
P value	ns	<0.05	<0.05

### Normal hepatocyte transplantation results in the clearance of DENA+RS-induced senescent hepa-tocytes

**Figure 3:**
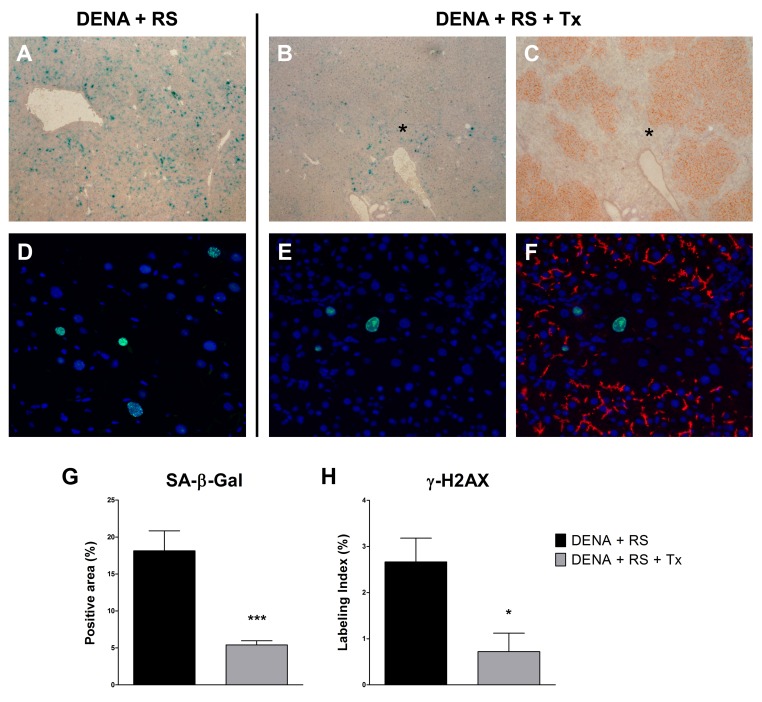
Hepatocyte transplantation reverses the RS-induced senescent phenotype Expression of SA-β-gal (panels **A, B, C** and **G**) and γ-H2AX (panels **D, E, F** and H), in rat liver exposed to either DENA+RS or DENA+RS followed by normal hepatocyte transplantations. Markers of cell senescence were highly expressed in DENA+RS-treated livers (panels **A, D**), while their levels were markedly reduced in animals receiving hepatocyte transplantation (panels **B, C, E, F**). In the latter group, extensive repopulation of the recipient liver was observed (panels **C**, histochemical staining for DPP-IV, orange-rust; panel **F**, immunofluorescence staining for CD26, red); note the residual expression of senescence markers in non-repopulated areas (panel **C** and **F**). Panels **A, B** and **C**: magnification 40x; panels **D, E** and **F**: magnification 200x. Panels **G** and **H**: ***P<0.001; *P<0.05.

As mentioned in the Introduction, recent findings have indicated that exposure to RS induces extensive hepatocyte senescence in rat liver [10]. Although cell senescence can represent a fail safe mechanism to alt neoplastic progression of altered cells [17], it is now well established that it can also contribute to the emergence of the neoplastic phenotype, possibly through secretion of a host of factors, variably referred to as senescence-associated secretory phenotype (SASP) [18] or senescence-messaging secretome (SMS) [19], and comprising cytokines, growth factors and proteases. Based on this information, it became important to determine the presence of hepatocyte senescence in animals treated with DENA+RS or DENA+RS+Tx. As reported in figure 3, markers related to cell senescence were highly expressed in animals treated with DENA+RS and killed 4 months later; these included the senescence-associated β-galactosidase (SA-β-gal), (panel A); and the phosphorylated form of H2A histone family, member X (γ-H2AX), which is considered as a marker of persistent activation of a DNA damage response and a trigger of cell senescence (panel D). However, both changes were almost completely reversed in animals given DENA+RS followed by hepatocyte transplantation. Transplanted hepatocytes were able to extensively repopulate the host liver; this effect was already prominent at 4 months post-injection (Figure 3, panel C), and persisted after 1 year (data not shown); it was associated with decreased expression of both SA-β-gal and γ-H2AX, which were virtually absent in repopulated areas of the liver and were only detected in residual portions of endogenous parenchyma (figure 3, panels B, C, E, F, G and H).

### Hepatocyte transplantation reverses biochemical markers of hepatocyte senescence and SASP

**Figure 4:**
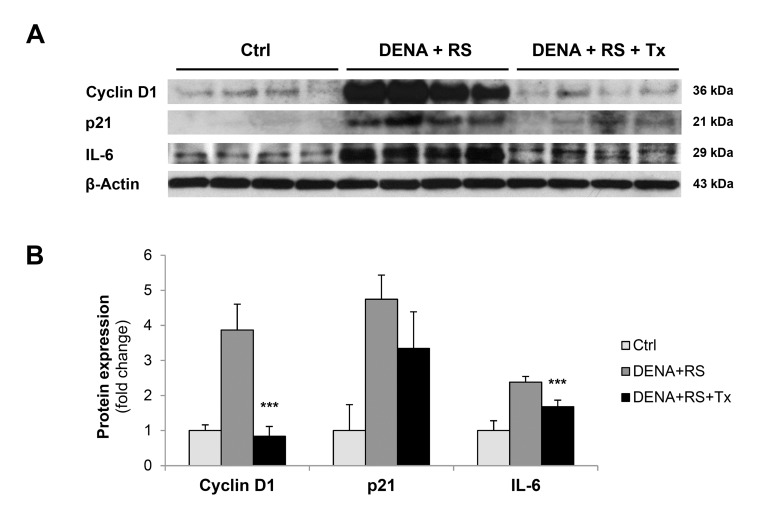
Hepatocyte transplantation reverses the RS-induced senescent phenotype Expression of cyclin D1, p21 and IL-6 in control rat liver and rat liver exposed to either DENA+RS or DENA+RS followed by normal hepatocyte transplantations. All gene products were highly expressed in DENA+RS-treated livers, while their levels were near control values in animals receiving hepatocyte transplantation (panels **A** and **B**). ***Significantly different from non-transplanted animals: P<0.005.

An intriguing interpretation of cell senescence postulates that this unique phenotype emerges when a cell integrates two types of signals: one that reads for growth and one that imposes a block in the replicative cycle [20,21]. For example, DNA damaging agents do not induce senescence in quiescent cells; however, they do so if the presence of persistent DNA damage and cell cycle arrest is coupled with growth promoting stimuli [21]. Under these conditions, cells switch on the senescence program and express markers related to both cell cycle block and growth stimulation. In line with this postulation, both the cyclin-dependent kinase inhibitor, p21, and the positive regulator cyclin D1 were found to be over-expressed in rats exposed to DENA+RS and killed 4 months thereafter (figure 4, panels A and B). Furthermore, a main component of SASP/SMS, namely the pro-inflammatory cytokine IL-6, was also over-expressed in DENA+RS-treated animals. Both findings were in agreement with those reported following exposure to RS alone [10].

Remarkably, these changes were strongly counteracted by transplantation of normal hepatocytes: in fact, the expression of p21, cyclin D1 and IL-6 returned to near-control levels in animals receiving normal cells following exposure to the carcinogenic protocol. (figure 4, panels A and B).

## DISCUSSION

The results of these studies indicate that transplantation of normal hepatocytes in a neoplastic-prone liver microenvironment delays the growth of hepatic nodules and the emergence of HCC; furthermore, this effect is associated with clearance of senescent hepatocytes induced by the carcinogenic protocol.

Over a decade ago, we reported that pre-neoplastic hepatocytes grew very rapidly and progressed to HCC upon transplantation into a host liver pre-treated with RS; however, the same cell population was unable to expand following implantation into the liver of a normal, un-treated recipient [8]. Recent studies, aimed at defining the biological and molecular determinants of the RS-induced effect, revealed the presence of extensive hepatocyte senescence in rat liver exposed to the alkaloid. Based on those findings, it was suggested that cell senescence and the associated SASP/SMS are possibly involved in the induction of the RS-associated neoplastic-prone tissue microenvironment [10]. In fact, it is now widely recognized that the senescence phenotype, while representing a fail-safe mechanism to avoid the risk of malignant transformation in cells harbouring damaged DNA or activated oncogenes [16,17,22,23], can also foster the emergence of premalignant and malignant cells [18,19,24,25], including their acquisition of metastatic potential [26] and resistance to chemotherapy [27,28]. These effects are at least partly mediated by a host of secreted factors, referred to as SASP/SMS and comprising cytokines, growth factors and proteases [18,19]. Among other products, the pro-inflammatory cytokine IL-6 has been attributed a prominent role both as a mediator of SASP effects and in reinforcing the senescence phenotype [24]. Moreover, cell senescence and SASP have been linked to chronic inflammation [29], adding yet another facet to the complex relationship between cancer, aging, and the immune response [30,31]. Interestingly, cell senescence has been reported in association with major risk factors for human neoplasia, including aging, cigarette smoke [32], UV light [33] and liver cirrhosis [34]. Indeed, the presence of hepatocyte senescence has long been documented during the evolution of chronic liver disease [35]. A recent study suggests that parameters related to cell senescence predict progression in non-alcoholic fatty liver disease (NAFLD) [36]. Moreover, a specific role for IL-6, together with TNF, has been proposed in the pathogenesis of liver inflammation and cancer associated with dietary and genetic obesity [37]. Thus, it appears that the tissue microenvironment induced by RS in rat liver, which strongly promotes the neoplastic process, shares intriguing similarities with chronic alterations associated with increased risk of liver cancer in humans.

In the present studies we tested the possibility that normal hepatocyte transplantation would reverse alterations induced by RS in the liver microenvironment, thereby modulating its tumour promoting potential. To this end, animals were sequentially exposed to DENA and RS, followed by two injections of hepatocytes freshly isolated from normal syngenic donors [14]. At end of 1 year, all animals treated with DENA+RS developed large liver tumours, with 50% (4/8) incidence of HCC. By contrast, the number of nodules were greatly reduced in rats receiving normal hepatocyte transplantation; most importantly, no animal in this group showed histological evidence of HCC (figure 2 and Table 1).

The liver of transplanted animals was extensively repopulated by donor-derived cells, resulting in the clearance of DENA+RS-induced senescent hepatocytes. Only residual hepatocyes expressing SA-β-Gal or γ-H2AX were found in these animals, and they were confined to areas of non-repopulated liver (figure 3); furthermore, the expression of cyclin D1, p21 and the SASP-associated cytokine IL-6 were markedly reduced to near control values. In summary, normal hepatocyte transplantation is able to delay DENA+RS-induced carcinogenic process and it is also associated with extensive remodeling of the tissue landscape, consisting in the massive replacement of resident senescent hepatocytes with phenotypically normal cells. It is noteworthy that our results are reminiscent of those reported by the group of the DeGregori in the hematopoietic system: it was observed that transplantation of young, normal bone marrow cells was able to prevent the clonal expansion and leukemogenesis mediated by initiated progenitors in the context of an aged or previously irradiated bone marrow microenvironment [38,39].

In a recent report, Kang et al. described the protective effect of immune-mediated clearance of N-ras-expressing senescent hepatocytes on liver cancer development in mice [40]. The effect was attributed to the putative preneoplastic nature of oncogene-transduced senescent cells, whose removal by a T-cell specific response was therefore considered as directly responsible for the reduced incidence of HCC [40]. While any direct involvement of the immune system was not investigated in our present study, our findings appear difficult to reconcile with the above proposition. In fact, there is no evidence that RS-induced senescent hepatocytes display any direct pre-neoplastic potential [41]; on the other hand, they are able to support the growth of transplanted nodular hepatocytes and their progression to HCC [8]. Thus, it appears that, under the conditions described in our studies, the role of cell senescence is to promote the growth of carcinogen-induced altered cells, possibly through the effect(s) of SASP/SMS components, including IL-6 [42]. Replacement of senescent hepatocytes by normal transplanted cells results in the attenuation of such promoting effect and a delay in the emergence of preneoplastic and neoplastic lesions. Interestingly, a similar paradigm could be applicable to the increased cancer incidence associated with aging [43].

Taken together, these findings reinforce the concept that strategies aimed at preserving and/or re-establishing a normal tissue microenvironment represent an effective approach towards limiting the impact of neoplastic disease. Furthermore, they highlight the role of senescent cells in fuelling carcinogenesis in a neoplastic-prone tissue landscape.

## EXPERIMENTAL PROCEDURES

### Animals and treatments

Liver carcinogenesis was induced using a sequential exposure to diethyl-nitrosamine (DENA) and retrorsine (RS) [14]. Male Fischer 344, rats of 4 weeks of age were injected with DENA (160 mg/kg, i.p.), followed by a single dose of RS (30 mg/kg, i.p.), given 10 days after DENA administration. Two weeks later, animals were divided into 2 groups of 12 rats each: group 1 received no further treatment, while group 2 was given two injections of hepatocytes isolated from a normal syngenic donor, containing 4×10^6^ cells each, two weeks apart. Animals from each group were killed at either 4 months (4 rats) or 12 months (the remaining 8 rats) after DENA administration. Starting 24 hours before killing, animals were given 3 injections of 5‘-bromo-deoxy-uridine (BrdU, 50 mg/kg, i.p.) every 8 hours. All experiments were approved by the University of Cagliari Ethical Committee for Animal Experimentation; all animals received humane care in accordance with NIH Guidelines for the care and use of animals. Hepatic lesions were microscopically classified according to published criteria [44].

### Hepatocyte isolation and transplantation

Hepatocytes for transplantation were isolated from a 6-wk old donor, according to a two-step collagenase perfusion technique [45]. Cell viability, determined by trypan blue exclusion at the end of the isolation procedure, was >90%. Animals were anesthetized and a small incision (about 1 cm) was performed in the upper abdominal wall; hepatocytes, suspended in PBS (1×10^7^/ml), were then delivered through a branch of the mesenteric veins, using a syringe with a 26-gauge needle. The fate of donor-derived cells in the recipient liver was followed using the F344-dipeptidyl-peptidase type IV (DPP-IV)-deficient model for cell transplantation [46]. Donor hepatocytes were isolated from animals expressing the marker enzyme (DPP-IV-positive), while DPP-IV-deficient rats were used as recipients. Since the Fischer 344 rat is a syngenic strain, no immunosuppression was required for successful cell transplantation.

### Histochemical and immunohistochemical methods

After sacrifice, livers were removed and samples were taken from each lobe to be either frozen for cryostat sections or fixed in buffered formalin for standard histological analysis and immunohistochemistry. In animals killed at 12 months, liver lobes were cut into 1-2 mm-thick slices and were macroscopically examined for the presence of hepatic nodules/tumors or any other evident lesion. The extent of liver repopulation in transplanted animals was monitored in cryostat sections stained for DPP-IV expression, using histochemical detection methods. Double staining for BrdU (DAKO, Glostrup, Denmark) and glutathione-S-transferase 7-7 (GST 7-7, Santa Cruz, Santa Cruz, CA) was performed on frozen sections, previously fixed in cold 1% acetic acid/ethyl alcohol and boiled in 0,01M Sodium Citrate, pH 6.0.

Staining for SA-β-gal was performed according to published procedures [47]. Immediately before staining, X-Gal stock solution was prepared by dissolving 20mg/ml X-Gal (Invitrogen, Carlsbed, CA) in dimetylformamide. SA-β-Gal staining solution was prepared as follows: 1 mg/ml of X-Gal stock solution were dissolved in 40 mM citric acid in sodium phosphate, pH 6.0/5 mM potassium ferrocyanide/5 mM potassium ferricyanide/150 mM NaCl/2 mM MgCl_2_. Frozen sections of 10-μm thickness were fixed for 5' in 4% formaldehyde/0.5% glutaraldehyde at 4°C, washed in PBS and incubated in fresh SA-β-Gal staining solution for 16h at 37°C. Sections were counterstained with Hematoxylin.

### Immunofluorescence

Immunoflorescence staining for γ-H2AX and CD26 was performed on frozen sections, following fixation in acetone. Slides were blocked for 30', incubated with primary antibodies (H2AX: Abcam, Cambridge, MA; CD26: BD Pharmigen, San Jose, CA) for 1 h at RT, then incubated with Alexa 488- and Alexa 555-conjugated secondary antibodies (Life Technologies, Carlsbad, CA). Slides were counterstained with DAPI and images were acquired with an IX71 fluorescence microscope with CCD camera (Olympus, Tokyo, Japan).

### Western Blot

Liver tissue samples were homogenized in RIPA lysis buffer containing Protease Inhibitors, then centrifuged at 12000 rpm for 30' at 4°C. Protein concentration in supernatants was measured using the BCA method [48]. Samples (20μg protein) were prepared in Laemmli buffer, boiled at 95°C for 5' then loaded into SDS-PAGE precast gels (Biorad, Hercules, CA) and run under denaturing conditions. Proteins were transferred onto nitrocellulose membranes (GE, Fairfield, CT), blocked with 5% non-fat milk for 1 h, then incubated with primary antibodies for Cyclin D1 (Sigma, St. Louis, MO), p21 (Santa Cruz, Santa Cruz, CA) NF-κB, TNF-α, IL-6 and β-Actin (Abcam) overnight at 4°C. Membranes were washed and incubated for 2 h with the appropriate secondary antibody conjugated with HRP. Protein bands were detected using a chemoluminescent substrate (Biorad) and imaged onto Kodak film.

### Imaging and Statistical analysis

Relative risk of developing preneoplastic/neoplastic lesions was calculated for both experimental groups, as shown in table 1. Chi-square test was used to evaluate statistical significance. Histological images and western blots were processed for quantification with Image Pro Premier software (Media Cybernetics, Rockville, MD). Results are presented as mean±S.E; two-tailed Student t test was used to evaluate results, with a lowest level of significance of p<0.05.
